# An Operative Manual

**Published:** 1874-07

**Authors:** 


*An Operative Manual. Ligation of Arteries.* By L. H. Farabeuf. Translated By John D. Jackson, M. D., of Danville, Ky. With Engravings. Philadelphia: J. B. Lippincott & Co., 1874. Buffalo: Martin Taylor.

It is to be regretted that in most of the Medical Schools of this country, so little attention is paid to the practical teaching of Operative Surgery. Beyond what they see in crowded clinics, medical students have but little knowledge of surgical procedures, few ever taking the trouble to undertake a course of operative surgery upon the cadaver. To those who are about to undertake such a course, the manual of Dr. Farabeuff, will be found to be an excellent guide, telling them not only what to do but how to do it.

It will be also an excellent manual for all who wish to perfect their knowledge of Operative Surgery, and will repay the student or practitioner who carefully consults its pages.

Dr. Jackson has performed his work well as a translator, and thanks are due to him for placing this manual in the hands of the American Profession. The work is amply illustrated; and the figures are all well made and fully explained. As a guide to the ligation of the arteries we do not know of a superior work.

